# Supplementary material for: Mutually Exclusive Expression of Closely Related Odorant-Binding Proteins 9A and 9B in the Antenna of the Red Flour Beetle Tribolium castaneum
Source: Biomolecules. 2021 Oct 12;11(10):1502. doi: 10.3390/biom11101502 (PMC8533528; doi:10.3390/biom11101502)
Supplement: Supplementary file 1 [file biomolecules-11-01502-s001.zip › biomolecules-1380979-supplementary.pdf]

Supplementary

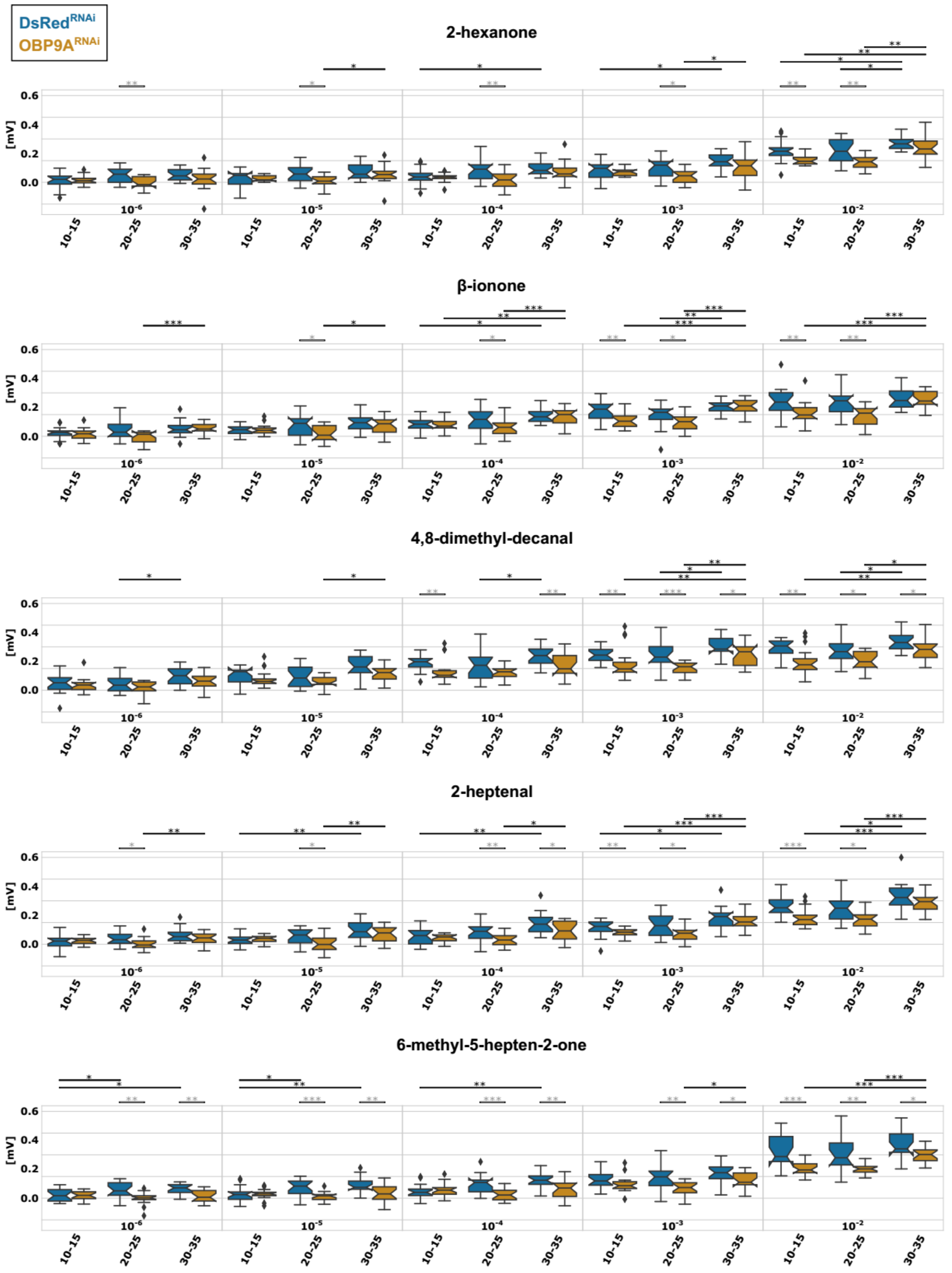

Figure S1. Peak EAG responses in mV of six ($N_{animals}$=6, $n_{replicates}$=3) female beetles to the olfactory stimulants 2-hexanone, β-ionone, 4,8-dimethyl-decanal, 2-heptenal, or 6-methyl-5-hepten-2-one after injection with dsRed (blue boxes) or TcasOBP9A (orange boxes) dsRNA and measured at three different post injections periods (10-15, 20-25, or 30-35 DPI). Statistical analysis between DsRed and OBP9A dsRNA (grey asterisk) or between different DPI (black asterisk) was performed per odor and dilution by Dunn's multiple comparison test, following initial Kruskal-Wallis test. Asterisks represent statistical significance levels (Holm corrected) of difference in median (* $p_{corr} < 0.05$, ** $p_{corr} < 0.01$, *** $p_{corr} < 0.001$).

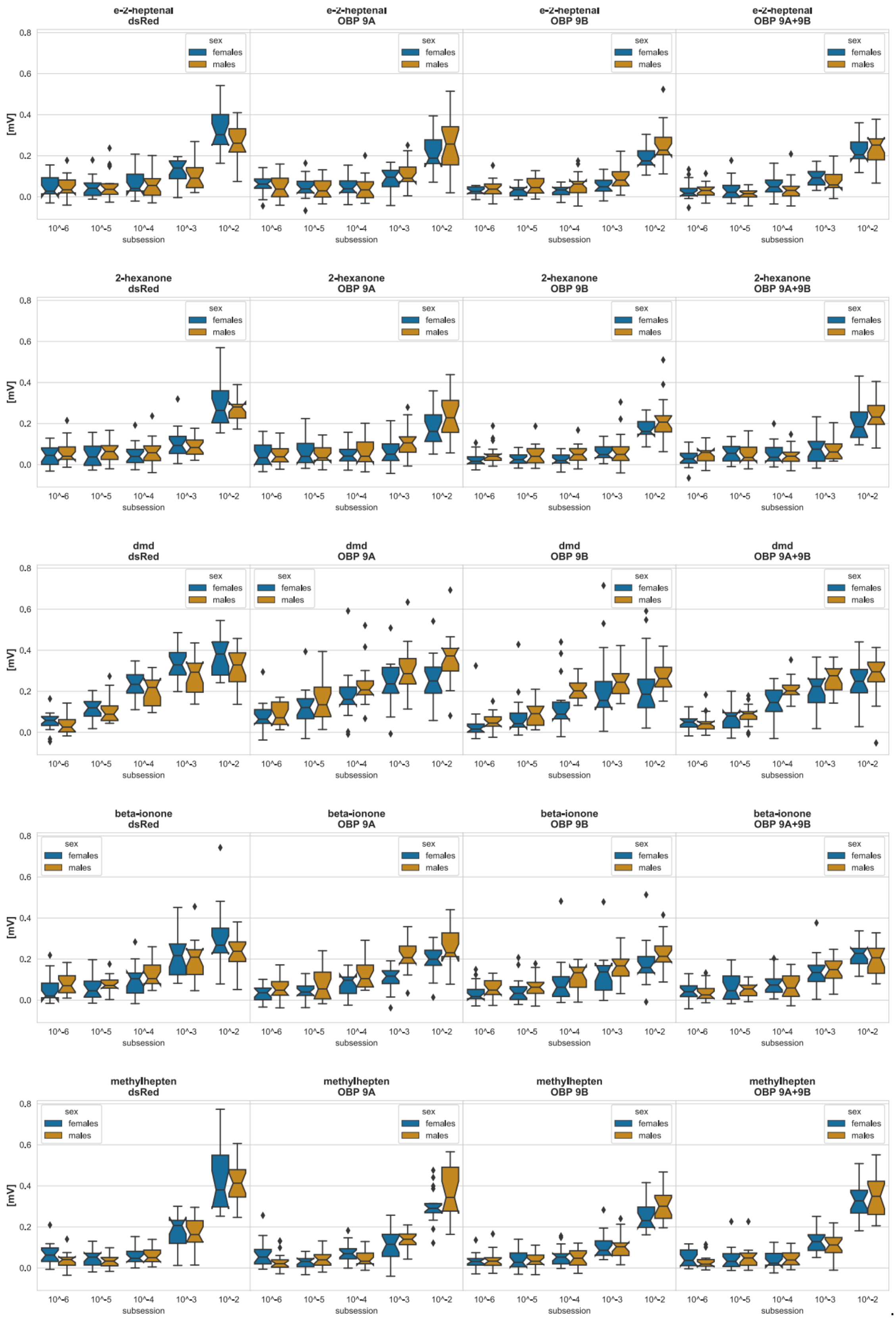

Figure S2. Peak EAG responses in mV of six male ($N_{animals}$ =6, $n_{replicates}$ =3; orange boxes) and six female ($N_{animals}$=6, $n_{replicates}$=3; blue boxes) beetles to the olfactory stimulants (E)-2-heptenal, 2-hexanone, 4,8-dimethyldecanal, β-ionone, or 6-methyl-5-hepten-2-one at 10-15 DPI with dsRed, TcasOBP9A, TcasOBP9B, or TcasOBP9A+9B dsRNA injection. The whiskers representing the 5–95% percentile of the peak amplitude EAG response in mV after robust LOESS smoothing and normalization to five log_{10} dilutions (10^{-2}–10^{-6}).

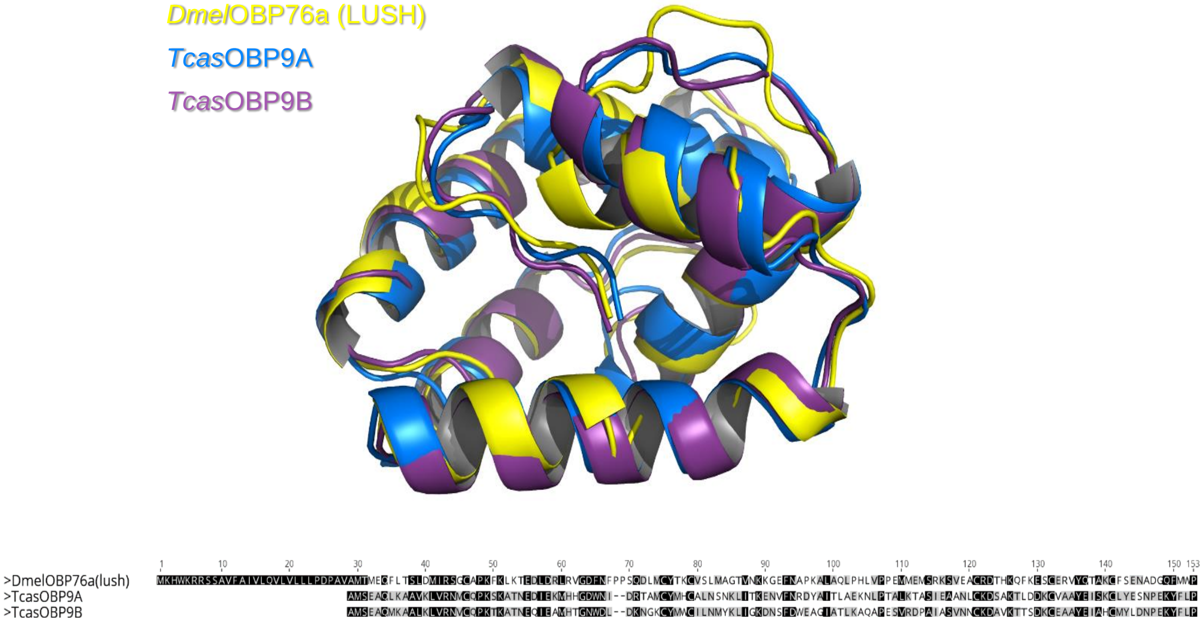

Figure S3. Structural superposition of the *Drosophila* OBP76a (Lush) with homology models of *Tcas*OBP9A and *Tcas*OBP9B depicted as cartoon. Below the corresponding sequence alignment is given.

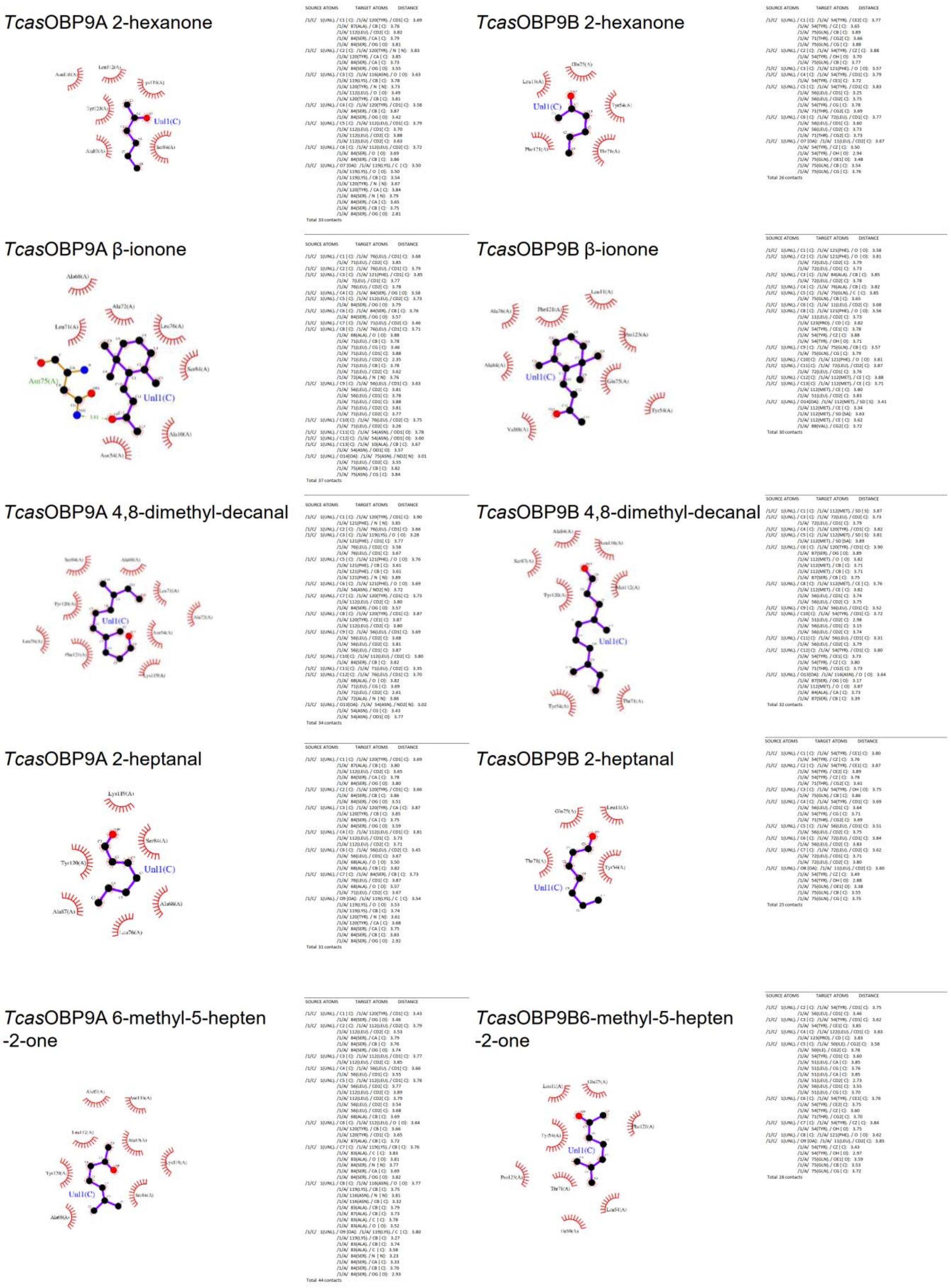

.

Figure S4. OBP-odor interactions. Two-dimensional (2D) ligand-protein interaction diagrams have been prepared for each docked ligand using the LigPlot+ software. The detailed list of inter-atomic distance between receptor atoms and docked ligands is listed on the right side of each 2D diagram (prepared with ncont program from the CCP4 suite).

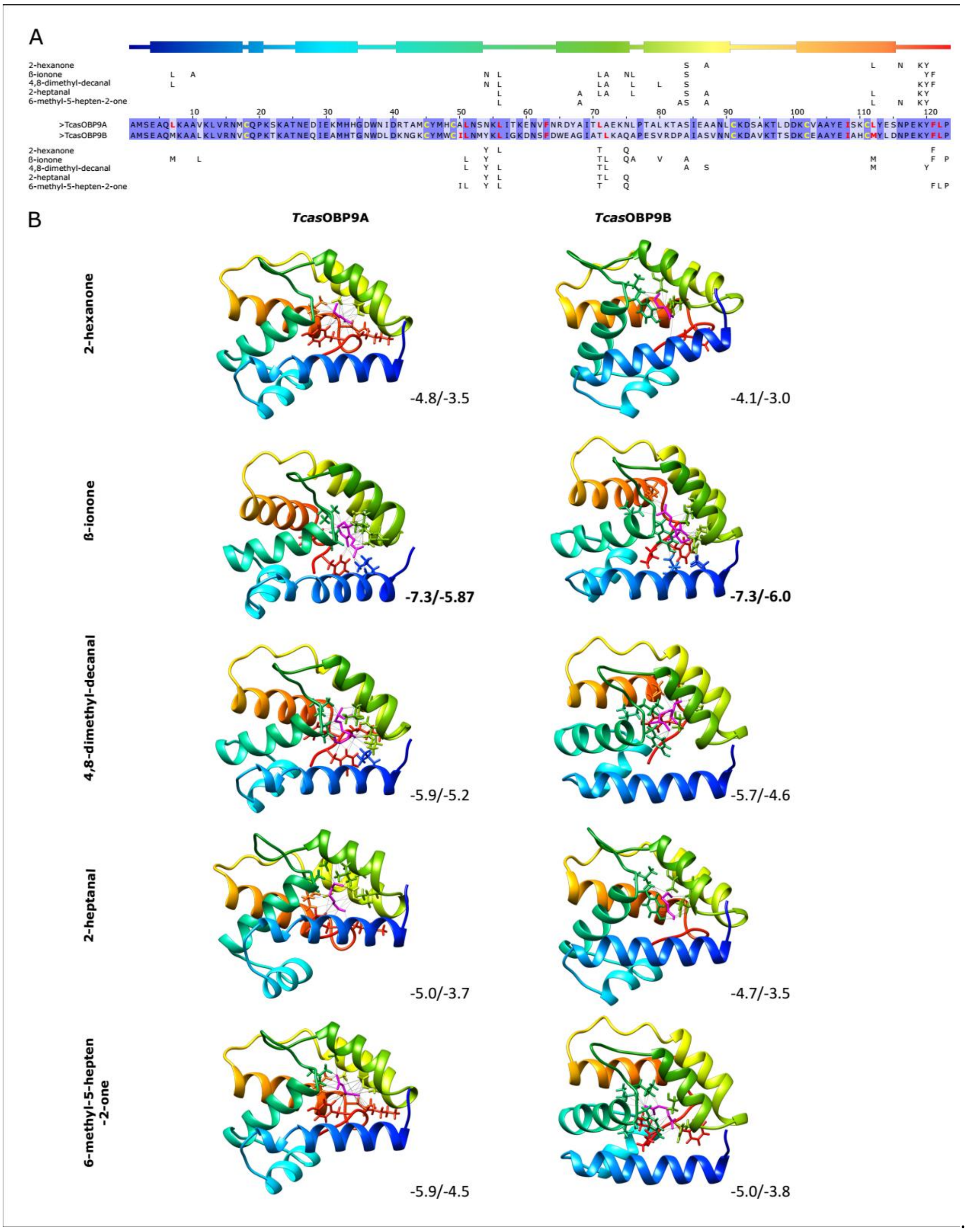

Figure S5. Odor-binding of TcasOBP9A and OBP9B (A) The domain structure of OBP9A with coloring corresponding to the rainbow ribbon model in B. Amino acid alignment of TcasOBP9A and TcasOBP9B as in Figure 4. Above and below of the alignment, the amino acid residues which are in predicted contact with the corresponding odor are depicted (based docking experiments with qvina and smina and prediction with Chimeras find clash/contacts). (B) Ribbon model of OBP9A and OBP9B binding the odors 2-hexanone, β-ionone, 4,8-dimethyl-decanal, 2-heptenal, or 6-methyl-5-hepten-2-one, respectively, based on modelling with ROSETTA (Song et al., 2013) [79] and visualized with Chimera (Pettersen et al., 2004) [93]. The residues that have contact to the respective odor are depicted as stick models, pseudo-bounds are shown in grey. Next to the models, the calculated affinities in kcal/mol (qvina/smina) are provided.

Supplementary List 1: List of species used for calculating the phylogenetic tree.

>*Amel*; *Apis mellifera* (Elsik et al., 2014; Foret and Maleszka, 2006) [48,49].
>*Hsal*; *Arpegnathos saltator* (Bonasio et al., 2010; McKenzie et al., 2014) [50,51].
>*Lhum*; *Linepithema humile* (Smith et al., 2011) [52].
>*Nvir*; Nasonia vitripennis (Vieira et al., 2012) [53].
>*Bmor*; *Bombyx mori* (Gong et al., 2009) [54].
>*Hmel*; *Heliconius melpomene* (Heliconius Genome Consortium et al., 2012) [55].
>*Dple*; *Danaus plexippus* (Zhan and Reppert, 2013) [56].
>*Gmor*; *Glossina morsitans* (Liu et al., 2010) [57].
>*Agam*; *Anopheles gambiae* (Lawson et al., 2009; Vieira and Rozas, 2011) [58,59].
>Dmel; *Drosophila melanogaster* (Consortium, 2003) [45].
>*Achi*; *Anoplophora chinensis* (Wang et al., 2017) [60].
>*Acor*; *Anomala corpulenta* (X. Li et al., 2015) [61].
>*Aqua*; *Ambrostoma quadriimpressum* (Wang et al., 2016) [62].
>*Blon*; *Brontispa longissimi* (Bin et al., 2017a) [63].
>*Cbow*; *Colaphellus bowringi* (X.-M. Li et al., 2015) [64].
>*Cchi, Callosobruchus chinensis* (Zhang et al., 2017) [65].
>*Cfor*; *Cylas formicarius* (Bin et al., 2017b) [66].
>*Dpon*; *Dendroctonus ponderosae* (Andersson et al., 2013; Keeling et al., 2013) [67,68].
>*Hobl, Holotrichia oblita* (Yin et al., 2019) [69].
>*Gdau*; *Galeruca daurica* (Li et al., 2017). [70]
>*Ldec*; *Leptinotarsa decemlineata* (Liu et al., 2015) [71].
>*Rfer*; *Rhynchophorus ferrugineus* (Yan et al., 2016) [72].
>*Tcas*; *Tribolium castaneum* (Dippel et al., 2014) [21].
>*Bger*; *Blattella germanica* (Robertson et al., 2018) [73].
>*Znev*; *Zootermopsis nevadensis* (Terrapon et al., 2014) [74].
